# IGF2BP2 Promotes Liver Cancer Growth Through an m6A-FEN1-Dependent Mechanism

**DOI:** 10.3389/fonc.2020.578816

**Published:** 2020-11-02

**Authors:** Jian Pu, Jianchu Wang, Zebang Qin, Anmin Wang, Ya Zhang, Xianjian Wu, Yi Wu, Wenchuan Li, Zuoming Xu, Yuan Lu, Qianli Tang, Huamei Wei

**Affiliations:** ^1^ Department of General Surgery, Affiliated Hospital of Youjiang Medical University for Nationalities, Guangxi, China; ^2^ Department of Hepatobiliary Surgery, Affiliated Hospital of Youjiang Medical University for Nationalities, Guangxi, China; ^3^ Graduate College of Youjiang Medical University for Nationalities, Guangxi, China; ^4^ Department of Pathology, Affiliated Hospital of Youjiang Medical University for Nationalities, Guangxi, China

**Keywords:** liver cancer, N6-methyladenosine, METTL3, IGF2BP2, FEN1

## Abstract

Hepatocellular carcinoma (HCC) is one of the most common malignant tumors in China. N6−methyladenosine (m6A) plays an important role in posttranscriptional gene regulation. METTL3 and IGF2BP2 are key genes in the m6A signal pathway and have recently been shown to play important roles in cancer development and progression. In our work, higher METTL3 and IGF2BP2 expression were found in HCC tissues and were associated with a poor prognosis. In addition, IGF2BP2 overexpression promoted HCC proliferation *in vitro* and *in vivo*. Mechanistically, IGF2BP2 directly recognized and bound to the m6A site on FEN1 mRNA and enhanced FEN1 mRNA stability. Overall, our study revealed that METTL3 and IGF2BP2, acting as an oncogene, maintained FEN1 expression through an m6A-IGF2BP2-dependent mechanism in HCC cells, and indicated a potential biomarker panel for prognostic prediction in liver cancer.

## Introduction

HCC is a highly lethal cancer (1). The estimated number of new patients that were diagnosed with HCC was about 900,000 and would result in approximately 800,000 deaths all over the world in 2018 (2). In China, the mortality and morbidity rates of HCC have demonstrated an increasing trend. Although medical treatment has been improved, the 5-year relative survival rate for HCC patients is still unsatisfactory. Therefore, identifying novel biomarkers and therapeutic targets for HCC diagnosis and treatment is an urgent need.

As one of the most prevalent modifications in eukaryotic messenger RNA (mRNA), N6-methyladenosine (m6A) affects almost every step of metabolism of messenger RNA (mRNA), such as mRNA processing, transport, translation, degradation and so on (3, 4). It is regulated by m6A modification enzymes (methyltransferases and demethylases) in a dynamically reversible manner (5, 6). In addition, there are also m6A-binding proteins, which can specifically bind to m6A and mediate their biological functions (7, 8). In recent years, m6A has been found to be associated with malignant tumors by more and more studies (9, 10). It has been reported to contribute to the self-renewal of tumor stem cells and promote the proliferation of malignant tumor cells. m6A is closely related to the phenotype and mechanism of malignant transformation, showing the possibility of m6A targeting therapy for human malignant tumors (10). In other words, m6A may become a new target for the treatment of malignant tumors. However, the functions of m6A modification and the underlying connections between the m6A methyltransferases, demethylases, and m6A-binding proteins are still unexplored in HCC.

Here, we first demonstrated the function of insulin-like growth factor 2 mRNA binding protein 2 (IGF2BP2) in facilitating HCC progression, and identified FEN1 as the downstream target of IGF2BP2. Overall, our study reveals that the METTL3-IGF2BP2-FEN1 axis is a potential therapeutic target for HCC.

## Materials and Methods

### Database and Bioinformatics

Level 3 gene expression profile (level 3 data) for HCC patients was obtained from the TCGA data portal (https://tcga-data.nci.nih.gov/tcga/). Differentially expressed genes (DEGs) were identified by Student’s t-test (Unpaired t test, two-tailed, ****P < 0.0001). The processed TCGA data is in **supplementary material**.

### Patient Samples

A total of 20 HCC tissues and 20 paired nontumorous liver samples were collected for qRT-CPR and IHC from the Affiliated Hospital of YouJiang Medical College for Nationalities from 2018 to 2019. The histological features of all specimens were evaluated by pathologists according to the standard criteria. The researchers were granted approval to conduct the research by Departmental Research Ethics Committee at the YouJiang Medical College for Nationalities. The study protocol was approved by the institutional review board of YouJiang Medical College for Nationalities. All the procedures were performed in accordance with the Declaration of Helsinki and relevant policies in China.

### m6A RNA Methylation Assay

Total RNA was extracted from samples using the Invitrogen life technologies TRIZOL. The change in m6A level relative to total mRNA was measured using EZ RNA Methylation Kit (ZYMO) following the manufacturer’s protocol. Each sample was analyzed using 200 ng of RNA isolated from different samples. Relative antification: To determine the relative m A RNA methylation status of two different RNA samples, a simple calculation for the percentage of m6A in total RNA was carried out using the following formula:

m6A%=(Sample OD−NCOD)/S×100%(PCOD−OD)/PS:is the amount of input sample RNA in ngP:is the amount of input positive control(PC)in ng.

### Immunohistochemistry

IHC was performed as described previously (11). The tissues were incubated with primary antibody against IGF2BP2 (ab128175, Abcam, 1:100) overnight in the refrigerator at 4°C. For negative controls, irrelevant primary antibodies were used. The corresponding secondary antibodies, conjugated to horseradish peroxidase (ab6721, Abcam, 1:1000), were incubated with the sections for 1 h at room temperature. After washing with PBS, the sections were incubated in horseradish enzyme-labeled chain avid in solution for 30 min at 37°C and washed again. The IHC score (0–9) was calculated by multiplying the intensity and the percentage scores (12).

### Western Blot Analysis

Tissues and cells were lysed in RIPA buffer (Beyotime, China). The lysates were denatured at 95°C for 5 min and then cooled down on ice. Then lysates were loaded on sodium dodecyl sulfatepolyacrylamide gel (SDS-PAGE) (10%) and electrotransferred onto polyvinylidene difluoride (PVDF) membrane. After blocking with 5% BSA blocking solution (SW3015, solarbio) for 1 h at room temperature, PVDF membranes were blotted with primary antibody at 4°C for 12 h, then incubated with HRP-labeled secondary antibody (CST, USA) at room temperature for 2 h. The bands were visualized using Tanon 5200 (Tanon, China). Primary antibodies are as follows: mouse monoclonal antibody to IGF2BP2 (ab128175, Abcam), rabbit monoclonal antibody to FEN1 (ab109132), and mouse monoclonal antibody to β-actin (CST, USA).

### Copy Number Variation (CNV) Detection Using the AccuCopy^®^ Assay

The AccuCopy assay (Shanghai, China) was used to evaluate the CNV of IGF2BP2. The basic molecular principle of AccuCopy is competitive PCR amplification. The primers used in this assay were:

Chr2-84500611-84500685(Reference gene): F: 5’- TGAGCCAAAAATTCAGAATACAAGGA -3’
R: 5’- TTGCTTGGAAGGCAGGCAAAC -3’
Chr16-25258413-25258537(Reference gene): F: 5’- GGGACAGGCCTGAAGTGTTTC -3’
R: 5’- AGCAGCAGCAGTGGGGTTTAG -3’
Chr3-185362000-185364000: F: 5’- CCGCAGACTTCTCATTCCTC -3’
R: 5’- GCAGCAGGAGCAGAAATACC -3’
Chr3-185367000-185369000: F: 5’- ATGGGCATTCATGTTTTGGT -3’
R: 5’- ACCCTGTGGTGATGGGATAA -3’

### Cell Culture and Transfection

HCC cell lines HepG2 and Huh-7 were gifts from obtained from Dr. C.M.W (HongKong University). MHCC97L and PLC were gifts from Dr. Z.Y. Tang (Fudan University). All cells were grown in a humidified incubator at 37°C with 5% CO_2_. shRNA that targets human IGF2BP2 or FEN1 (psi-LVRU6MP- IGF2BP2) and the scrambled shRNA were purchased from GeneCopoeia (Rockville, MD, USA). The following sequences were targeted to human IGF2BP2 or FEN1:

IGF2BP2: 5’- GCATATACAACCCGGAAAGAA-3’.
FEN1: 5’- GATGCCTCTATGAGCATTTAT-3’

### Cell Proliferation and Colony Formation Assay

For cell proliferation assays, CCK-8 kit (Dojindo, Kumamoto, Japan) and EdU Apollo^®^ 567 In Vitro Imaging Kit (Ribobio, Guangzhou, China) were used according to the manufacturer’s instruction. For the colony formation assay, 2×10^3^ cells were plated into six-well plate, and cultured in complete culture medium. After 10 days, colonies were fixed with 4% paraformaldehyde and stained with 0.1% crystal violet (Beyotime, Beijing, China). Finally, visible colonies were photographed (Nikon, Tokyo, Japan) and counted. All experiments were performed in triplicate.

### MeRIP-Seq and RNA-Seq

50 μg of total RNA was extracted and purified using PolyTtract mRNA Isolation System (Promega, Hong Kong). After fragmentation, RNA was incubated with an anti-m6A antibody for one hour at 4°C, and then mixed with prewashed Protein A/G Magnetic Beads (Merck Millipore, Germany) in immunoprecipitation buffer at 4°C overnight. Enrichment of m6A containing mRNA was purified for further MeRIP sequencing by RiboBio (Guangzhou, China). RNA-seq was conducted in accordance with a previously reported protocol (13). Fold change of >1.5 and false discovery rate P < 0.05 were set as the cutoffs to screen for differentially expressed genes (DEGs).

### RIP-qPCR

RIP was conducted with the Magna RIP RNA-Binding Protein Immunoprecipitation Kit (Merck Millipore, Germany) according to the manufacturer’s protocol. Mouse immunoglobulin G or IGF2BP2 (ab128175, Abcam) were coated with Magnetic beads. Then the coated beads were incubated with prepared cell lysates overnight at 4°C. Then, the RNA was finally extracted. The relative interaction between FEN1 and IGF2BP2 transcripts was determined by qPCR and normalized to the input.

### Animals

The animal studies were approved by the Institutional Animal Care and Use Committee of Affiliated Hospital of YouJiang Medical College for Nationalities, and were carried out according to institutional guidelines. A total of 40 BALB/c nude mice were chosen and assigned to two groups: shCtrl group (injected with HepG2 cells) and shIGF2BP2 group (injected with HepG2 cells with IGF2BP2 knockdown). 200 µl of the above cell suspension containing 2 × 10^5^ cells was injected into the left or right back of each mice. Tumor sizes and tumor volume were measured as described previously (14).

## Results

### Elevated METTL3 and IGF2BP2 Expression Correlate With Poor Prognosis of Patients With HCC

**Figure 1:**
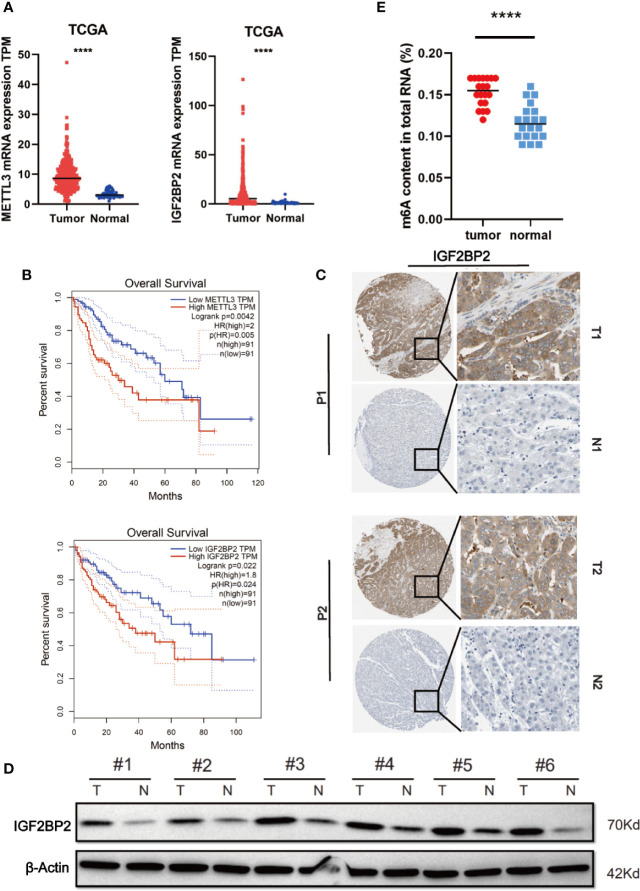
Elevated IGF2BP2 expression correlates with poor prognosis of patients with HCC. **(A)** METTL3 (left) and IGF2BP2 (right) mRNA levels in the TCGA databases. **(B)** Kaplan-Meier survival curves of OS based on METTL3 and IGF2BP2 expression using the online bioinformatics tool GEPIA. **(C)** Representative IHC images of IGF2BP2 protein expression in HCC tissues (T) and adjacent normal tissue (N) of two patients (P1–P2). **(D)** IGF2BP2 protein levels were measured in HCC tissues and paired normal tissues by western blotting. **(E)** An m6A RNA methylation assay revealed the m6A content in HCC tissues and adjacent normal tissues. (****P < 0.0001, Student’s t-test).

To elucidate the functional roles of m^6^A modification in HCC, we investigated the expression of 20 genes involved in m^6^A RNA modification in the TCGA-LIHC databases (15). The results revealed that METTL3 and IGF2BP2 were significantly upregulated in tumor tissues compared with those in adjacent normal tissues (**Figure 1A**). Utilizing the online bioinformatics tool GEPIA (http://gepia.cancer-pku.cn/detail.php) (16), we also found that patients with HCC with increased METTL3 and IGF2BP2 mRNA levels had worse overall survival (OS) (**Figure 1B**) (the group cutoff was set as quartile [cutoff-high (%) was 75% and cutoff-low (%) was 25%)]. This result was consistent with previous reports that the METTL3 expression was up-regulated in the tumor tissue of liver cancer (17). To further validate the expression level of IGF2BP2 in the real world, we tested the expression of IGF2BP2 protein in 20 patients’ HCC tissues and adjacent normal tissues by IHC. The results displayed that the high expression of IGF2BP2 was detected in 13/20 (65%) HCC (**Figure 1C**). Higher expression of IGF2BP2 was also found in HCC tissues than in the corresponding adjacent nontumor HCC tissues by western blot analysis (**Figure 1D**). Furthermore, statistical analysis of the 20 HCC patients’ tumor tissues showed that expression of IGF2BP2 was associated with tumor size (p = 0.031) (**Table 1**). We then examined the m6A RNA levels in 20 HCC tissues and paired adjacent tissues by m6A RNA methylation assay. We found that the m6A RNA levels were significantly higher in HCC tissues than paired adjacent tissues (**Figure 1E**). Thus, the above results reveal that the m6A modification and the expression of IGF2BP2 are increased in HCC and that IGF2BP2 might be an independent prognostic factor for patients with HCC.

**Table 1:** Relationship between IGF2BP2 expression in patients with HCC and clinicopathologic characteristics.

Feature	No.	YTHDF1 expression		P value
		High	Low	
Age (years)				
≥60	12	5	7	0.142
<60	8	6	2	
Tumor size(cm)				
≥2.0	13	9	4	0.019^*^
<2.0	7	1	6	
Lymph node metastasis				
Yes	12	6	6	0.582
No	8	5	3	
Clinical Stage				
I/II	6	2	4	0.202
III/IV	14	9	5	

(The P value was calculated by chi-squared analysis). *P < 0.05.

### DNA Copy Number Aberrations Promote IGF2BP2 Overexpression in HCC

**Figure 2:**
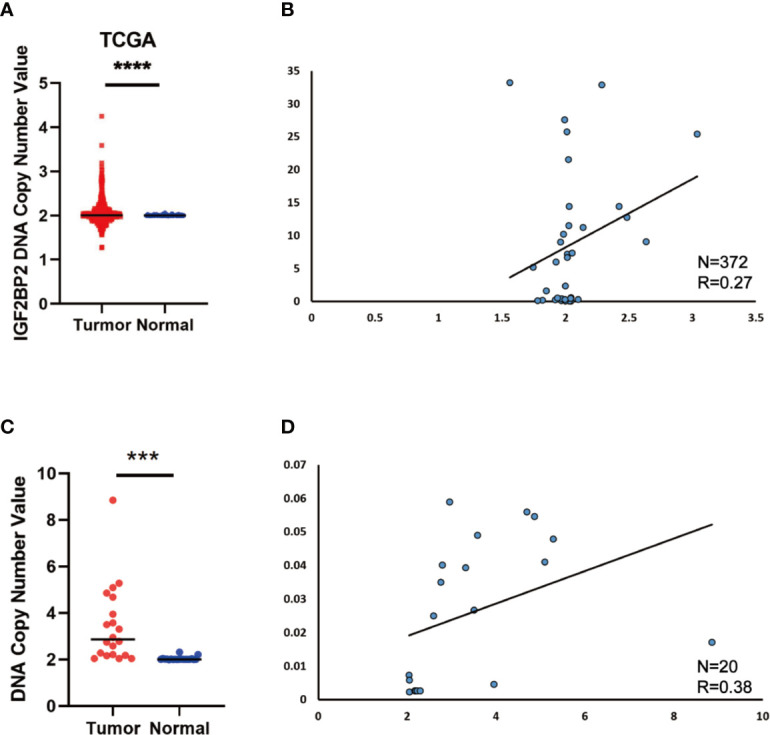
DNA Copy Number Aberrations Promote IGF2BP2 Overexpression in HCC. **(A)** IGF2BP2 copy numbers in the TCGA databases. **(B)** Correlation between the DNA copy number and mRNA expression of IGF2BP2 in TCGA. **(C, D)** The comparison of the IGF2BP2 DNA copy number between HCC tissues (T) and adjacent normal tissues (N) in 20 patients and the correlation between the DNA copy number and mRNA expression of IGF2BP2 in 20 HCC tissue. (****P < 0.0001, ***P < 0.001, Student’s t-test).

Since the copy number amplification (CNA) is closely related with gene expression. To detect whether DNA CNA contributes to IGF2BP2 overexpression or not, we extracted the CNA and expression data of IGF2BP2 from TCGA. After analyzing the data, we identified that IGF2BP2 copy number in HCC tissues was significantly higher than that in normal tissues (**Figure 2A**). Then, we evaluated the correlation of the CNA with the expression of IGF2BP2 by Pearson correlation coefficient [32]. The results showed that the expression of IGF2BP2 was significantly associated with the CNA in TCGA-LIHC samples (**Figure 2B**, P <0.01). To further verify the association between the expression and CNA, the AccuCopy copy number assay (18) was applied to validate 20 pairs of primary HCC tissues compared with adjacent normal tissues. We discovered that the copy number of IGF2BP2 in tumor tissues was higher than that in adjacent normal tissues (**Figure 2C**). Additionally, the CAN was also correlated with mRNA expression of IGF2BP2 in the real world *via* qRT-PCR (**Figure 2D**, P <0.05). Therefore, our results proved that CNA was one of the mechanisms that contributed to the overexpression of IGF2BP2 in HCC.

### IGF2BP2 Promotes HCC Cells Tumorigenicity *In Vitro* and *In Vivo*

**Figure 3:**
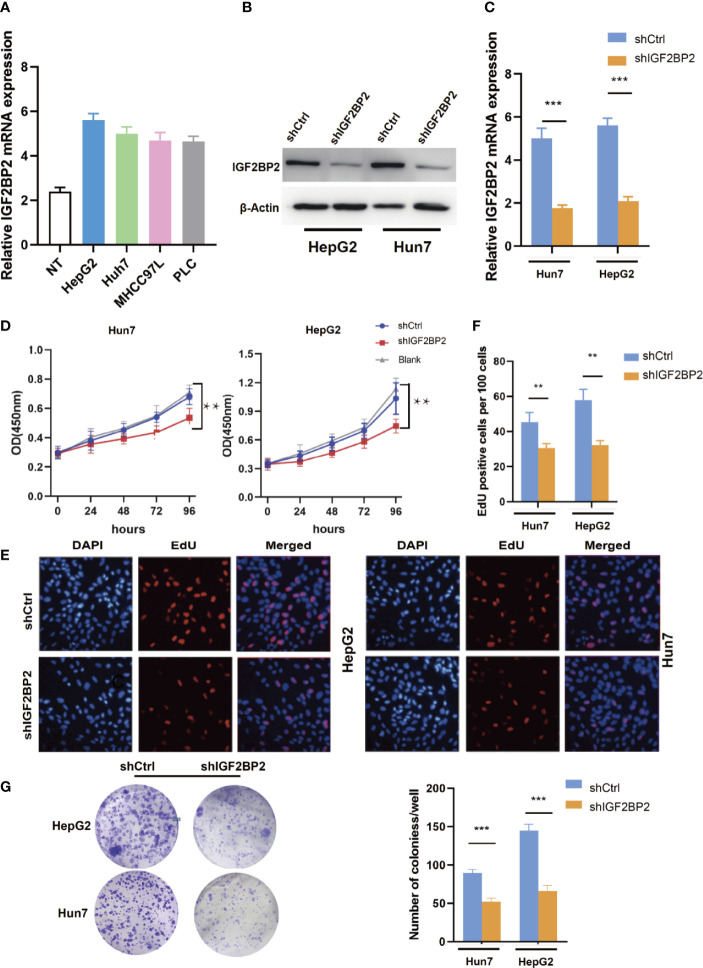
IGF2BP2 Promotes HCC Cells Tumorigenicity *in vitro*. **(A)** The expression of IGF2BP2 mRNA in HCC cells was determined using qRT-PCR (The experiment was repeated three times). **(B, C)** Expression level of IGF2BP2 knockdown efficiency in HepG2 and Hun7 cell lines was detected by western blot and qRT-PCR. **(D)** The influences of IGF2BP2 knockdown on cell proliferation were confirmed using the CCK-8 assay (**P < 0.01, Student’s t-test). **(E, F)** The influences of IGF2BP2 knockdown on cell proliferation were confirmed by EdU assay (**P < 0.01, Student’s t-test). **(G)** The representative picture of colony formation assay, and the quantification of colonies per well (***P < 0.001, Student’s t-test).

**Figure 4:**
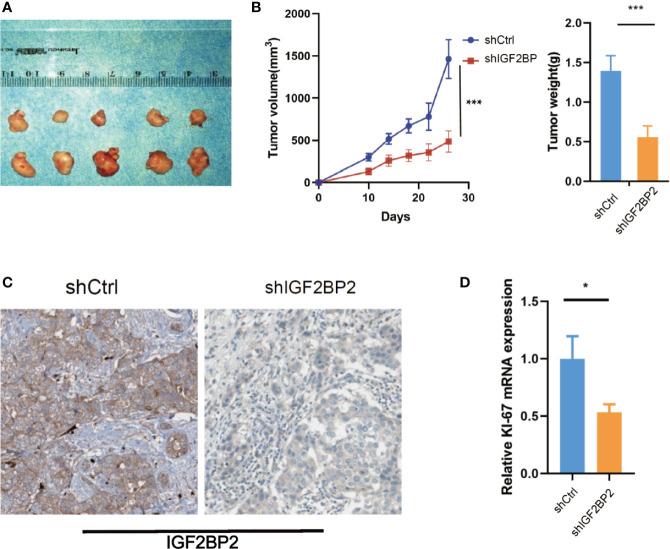
IGF2BP2 Promotes HCC Cells Tumorigenicity *in vivo*. **(A, B)** Knockdown of IGF2BP2 effectively inhibited HepG2 cells subcutaneous tumor growth in nude mice (***P < 0.001, Student’s t-test). **(C)** IHC analysis of IGF2BP2 in tumor tissue samples. **(D)** qRT-PCR analysis of KI-67 in tumor samples. (*P <0.05, Student’s t-test).

To select suitable cell lines for *in vitro* experiments, the IGF2BP2 expression was detected by qRT-PCR. Consistent with results in tumor tissue, the IGF2BP2 expression was significantly up-regulated in HCC cell lines (**Figure 3A**). Then, we selected HepG2 and Hun7 cell lines to investigate the the function of IGF2BP2 *in vitro*. To confirm the oncogenic function of IGF2BP2, shRNAs specifically targeting IGF2BP2 were transfected into HepG2 and Hun7 cells by lentivirus infection, respectively. The silencing effect was determined by western blot and qRT-PCR analysis, and the results showed that shRNA specifically downregulated the expression of IGF2BP2 (**Figures 3B, C**). The influence of IGF2BP2 knockdown on cell proliferation was examined by using the CCK-8 and EdU proliferation assays. CCK-8 and EdU analysis suggested that cell proliferation in HepG2 and Hun7 cell lines was impeded after IGF2BP2 knockdown (**Figures 3D–F**). Furthermore, knockdown the expression of IGF2BP2 reduced growth ability owing to fewer colonies formed after 10 days than the shCtrl group in both cell lines (**Figure 3G**). To determine if knockdown the IGF2BP2 expression could reduce tumor growth *in vivo*, normal IGF2BP2 expression and knockdown HepG2 cells were transfected into the animals, respectively. The tumor growth was monitored. As shown in **Figures 4A, B**, knockdown the IGF2BP2 expression reduced tumor growth, as evidenced by the higher tumor volume and tumor weight; IGF2BP2 expression could not be detected in tumor tissues of IGF2BP2 knockdown mice (**Figure 4C**). Tumor tissues from knockdown IGF2BP2 mice showed lower levels of KI-67, as compared to control mice (**Figure 4D**). Together, the findings above suggested that the IGF2BP2 expression promoted HCC cell proliferation *in vitro* and *in vivo*.

### FEN1 Is Regulated by METTL3-Mediated m6A Modification and Recognized by IGF2BP2 *via* an m6A-Dependent Manner

**Figure 5:**
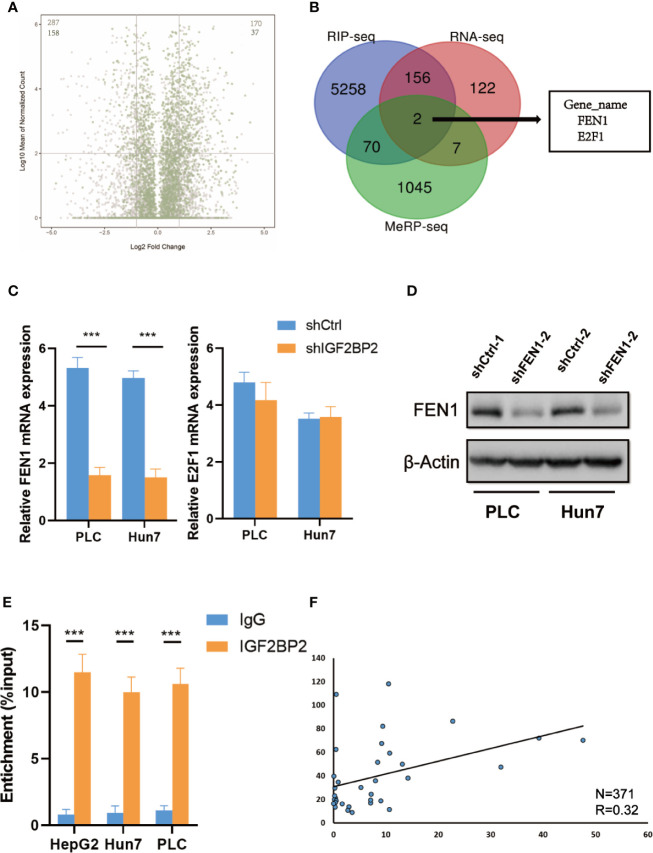
FEN1 is regulated by METTL3-mediated m6A modification and recognized by IGF2BP2 *via* an m6Adependent manner. **(A)** Volcano plots displaying enrichment of dysregulated target genes in IGF2BP-knockdown (shIGF2BP) versus control (shNS) HepG2 cells. The numbers of significantly downregulated or upregulated genes (|log2 FC| > 1, P < 0.01, two-tailed Student’s t-test) in the RIP-seq target group. **(B)** RNA-seq, MeRIP-seq and RIP-seq identified differentially expressed genes in HepG2 cells when compared with their corresponding controls. **(C)** Relative changes in FEN1 and E2F1 mRNA levels upon IGF2BP2 silencing in other HCC cell lines (***P < 0.001, Student’s t-test). **(D)** The protein levels of FEN1 in other HCC cell lines were measured by western blotting. **(E)** RIP–qPCR showing the binding of IGF2BP2 to FEN1 (***P < 0.001, Student’s t-test). **(F)** IGF2BP2 expression was positively correlated with FEN1 expression in HCC in TCGA database.

To map target transcripts by which IGF2BP2 promotes HCC progression, we first performed immunoprecipitation sequencing (MeRIP-seq) in HepG2 cells with stable METTL3 knockdown. We identified 1124 m6A modified genes in HepG2 cells (fold change of >1.2). We next conducted RNA sequencing (RNA-seq) in IGF2BP2 knockdown HepG2 cells. In addition, we analyzed RIP–seq data in HepG2 cells from the GEO database (19). We categorized transcripts into three groups: non-m6A marked transcripts, m6A-containing transcripts, and m6A-marked transcripts bound by IGF2BP2. Knockdown of IGF2BP2 globally and preferentially inhibited the expression of RIP targets (**Figure 5A**). Intriguingly, two genes were overlapped in the RNA-seq, MeRIP-seq, and RIP-seq data, and they were E2F1 and FEN1 (**Figure 5B**). Next, we validated the mRNA levels of these two candidate genes in other IGF2BP2 knockdown HCC cell lines *via* qRT-PCR (Huh7 and PLC). Only FEN1 but not E2F2 was consistently regulated by IGF2BP2 in all three HCC cell lines (**Figure 5C**). We also confirmed *via* a western blot assay that the FEN1 protein levels were positively regulated by IGF2BP2 in different HCC cell lines (**Figure 5D**). Next, RIP-qPCR demonstrated a strong binding of IGF2BP2 with FEN1 in all three HCC cell lines (**Figure 5E**). We also found that the expression of IGF2BP2 was significantly correlated with FEN1 expression in the TCGA-LIHC database (**Figure 5F**). Together, our experiments indicate that METTL3-mediated m6A modification maintains FEN1 expression *via* IGF2BP2-dependent FEN1 mRNA stability.

### FEN1 Plays an Oncogenic Role in Ovarian Cancer Cells in HCC

**Figure 6:**
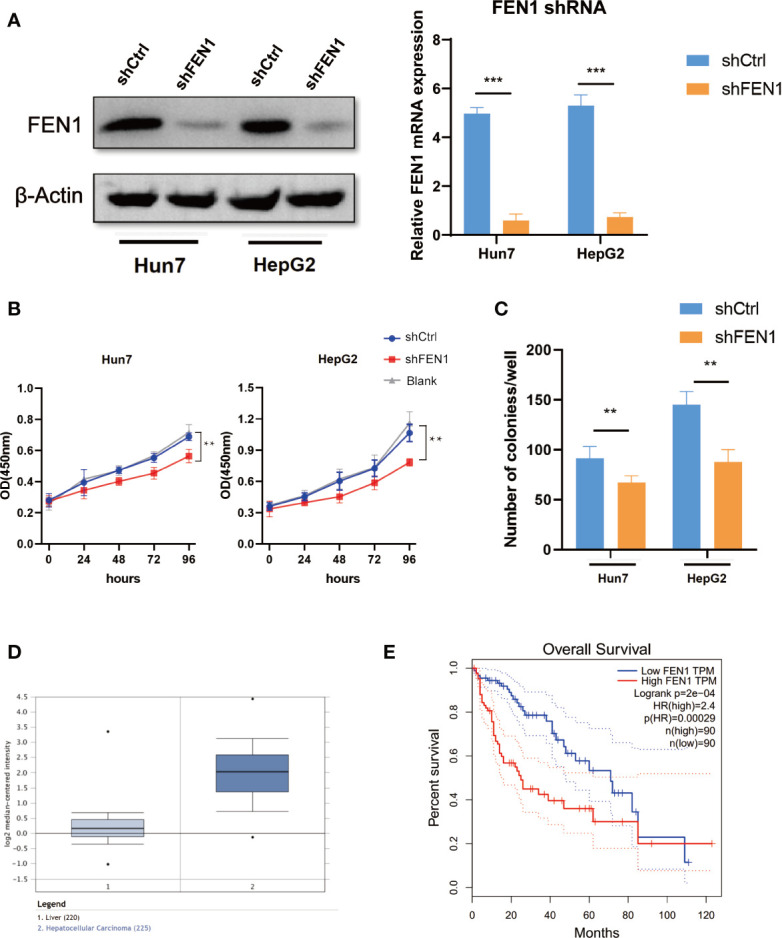
FEN1 plays an oncogenic role in ovarian cancer cells in HCC. **(A)** Expression level of FEN1 knockdown efficiency in HepG2 and Hun7 cell lines was detected by western blot and qRT-PCR (***P < 0.001, **P < 0.01, Student’s t-test). **(B)** The influences of FEN1 knockdown on cell proliferation were confirmed using the CCK-8 assay. **(C)** The influences of FEN1 knockdown on the quantification of colonies per well. **(D)** FEN1 mRNA levels in the oncomine databases. **(E)** Kaplan-Meier survival curves of OS based on FEN1 expression using the online bioinformatics tool GEPIA.

To further examine the role of FEN1 in HCC, we analyzed the cellular phenotypes in HepG2 and Hun7 cells, including cell growth and colony formation ability upon FEN1 knockdown. shRNA specifically targeting FEN1 was transfected into HepG2 and Hun7 cells by lentivirus infection, respectively. The silencing effect was determined by western blot and qRT-PCR analysis, and the results showed that shRNA specifically downregulated the expression of FEN1 (**Figure 6A**). Notably, cell growth and colony formation capacities were markedly suppressed upon FEN1 knockdown in both HepG2 and Hun7 cells (**Figures 6B, C**). Based on the mechanism we identified above, we proceeded to explore the clinical relevance of FEN1. Utilizing the online bioinformatics tool oncomine (https://www.oncomine.org/), we found that FEN1 was significantly upregulated in tumor tissues compared with that in normal tissues (**Figure 6D**). Moreover, we also found that patients with HCC with increased FEN1 mRNA levels had worse overall survival (OS) (**Figure 6E**) (The group cutoff was set as Quartile [cutoff-High (%) was 75% and cutoff-Low (%) was 25%)].

## Discussion

HCC is one of the most common cancers and the leading cause of cancer-related mortality in developing countries (20). However, the molecular pathogenesis of HCC remains largely unknown (21, 22). Therefore, better prognostic indicators are needed to identify patients with poor prognosis and intervene as early as possible.

m6A, the most abundant post-transcriptional modification, is mainly regulated by m6A WERs (“writers”, “erasers,” and “readers”) in diverse cell types (23, 24). m6A can affect the stability of mRNA *via* the m6A-binding protein IGF2BPs (IGF2BP1, IGF2BP2, and IGF2BP3) (19, 25). Dysregulation of m6A pathway components could affect oncogene expression, thereby linking m6A with tumorigenesis (26–28). In the present work, by utilizing the TCGA database, we identified that the expression of METTL3 and IGF2BP2 was upregulated in HCC tissues and that the high expression was correlated with poor prognosis. Our data with the clinical HCC samples were similar to the online data, in which IGF2BP2 was upregulated in HCC tissues at both the mRNA and protein levels, and the CNA might promote the high expression of IGF2BP2 in HCC. Moreover, the IGF2BP2 expression was found to be correlated with tumor size. But note that the clinical HCC specimens were just 20 pairs, further clinical trials in a multicenter are needed.

Flap endonuclease-1 (FEN1) is a multifunctional, structure-specific nuclease that has a critical role in maintaining human genome stability (28). Regulatory mechanisms of FEN1 in cells are crucial to maintaining normal cell growth (29). He et al. compared the expression of FEN1 in cancer tissues and normal tissues, and found that the expression of FEN1 in lung cancer tissues was significantly increased (30). In addition, high expression of FEN1 was also found in breast cancer and gastric cancer (31, 32). However, the mechanism of FEN1 up regulation is still unclear. In our study, m6A-seq and RNA-seq revealed that FEN1 was the downstream gene of IGF2BP2 and confirmed the oncogenic effect of FEN1 and revealed an m6A-dependent regulatory mechanism to partially explain the common upregulation of FEN1 in cancer.

In conclusion, our study identified that CAN was the major mechanism that promoted the high expression of IGF2BP2 in HCC. Additionally, the panoramic network of “writer” METTL3, “reader” IGF2BP2, and “target” FEN1 emphasized a novel m6A-dependent gene regulatory biological process. Our study suggests that targeting METTL3-IGF2BP2-FEN1 may be a novel and efficient strategy for a tumor-targeting therapy for HCC.

## Data Availability Statement

The raw data supporting the conclusions of this article will be made available by the authors, without undue reservation.

## Ethics Statement

The study protocol was reviewed and approved by the institutional review board of YouJiang Medical College for Nationalities. All the procedures were performed in accordance with the Declaration of Helsinki and relevant policies in China. The patients/participants provided their written informed consent to participate in this study. The animal studies were reviewed and approved by the Institutional Animal Care and Use Committee of Affiliated Hospital of YouJiang Medical College for Nationalities.

## Author Contributions

Methodology: ZQ, AW, and YZ. Grammar Correction: YW and WL. Sample Collection: QT. All authors contributed to the article and approved the submitted version.

## Funding

This work was supported by Science and Technology Project, Guangxi, AD17129025.

## Conflict of Interest

The authors declare that the research was conducted in the absence of any commercial or financial relationships that could be construed as a potential conflict of interest.
