# Supplementary figures and images for: IGF2BP2 Promotes Liver Cancer Growth Through an m6A-FEN1-Dependent Mechanism
Source: Front Oncol. 2020 Nov 2;10:578816. doi: 10.3389/fonc.2020.578816 (PMC7667992; doi:10.3389/fonc.2020.578816)

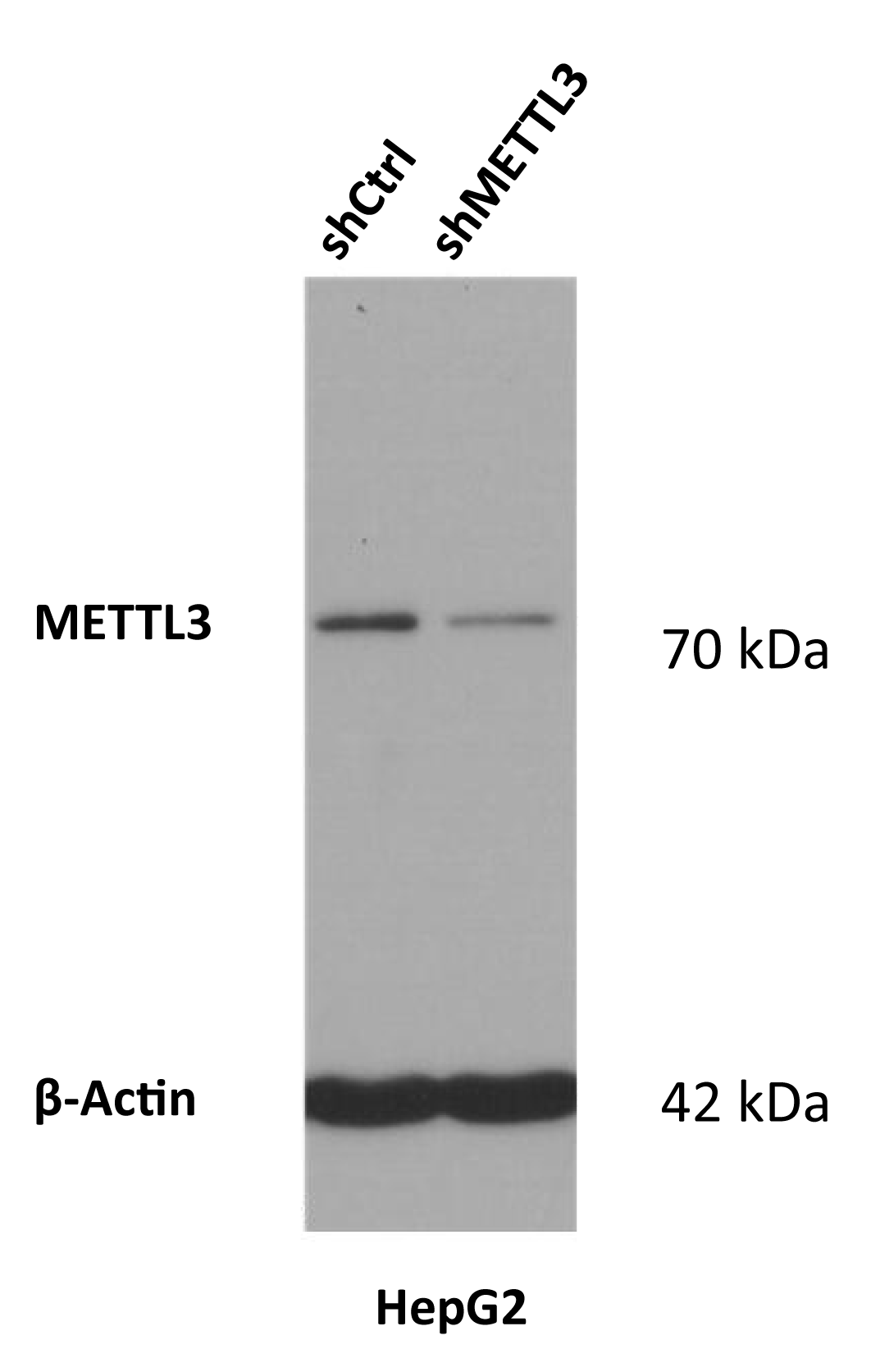

Supplement: Supplementary file 1 [file Image_1.tif]
